# Comparative Study of Transcriptomics-Based Scoring Metrics for the Epithelial-Hybrid-Mesenchymal Spectrum

**DOI:** 10.3389/fbioe.2020.00220

**Published:** 2020-03-20

**Authors:** Priyanka Chakraborty, Jason T. George, Shubham Tripathi, Herbert Levine, Mohit Kumar Jolly

**Affiliations:** ^1^Centre for BioSystems Science and Engineering, Indian Institute of Science, Bengaluru, India; ^2^Center for Theoretical Biological Physics, Rice University, Houston, TX, United States; ^3^Medical Scientist Training Program, Baylor College of Medicine, Houston, TX, United States; ^4^Ph.D. Program in Systems, Synthetic, and Physical Biology, Rice University, Houston, TX, United States; ^5^Department of Physics, College of Science, Northeastern University, Boston, MA, United States; ^6^Department of Bioengineering, College of Engineering, Northeastern University, Boston, MA, United States

**Keywords:** EMT, MET, EMT score, EMT quantification, tumor heterogeneity, hybrid epithelial/mesenchymal

## Abstract

The Epithelial–mesenchymal transition (EMT) is a cellular process implicated in embryonic development, wound healing, and pathological conditions such as cancer metastasis and fibrosis. Cancer cells undergoing EMT exhibit enhanced aggressive behavior characterized by drug resistance, tumor-initiation potential, and the ability to evade the immune system. Recent *in silico*, *in vitro*, and *in vivo* evidence indicates that EMT is not an all-or-none process; instead, cells can stably acquire one or more hybrid epithelial/mesenchymal (E/M) phenotypes which often can be more aggressive than purely E or M cell populations. Thus, the EMT status of cancer cells can prove to be a critical estimate of patient prognosis. Recent attempts have employed different transcriptomics signatures to quantify EMT status in cell lines and patient tumors. However, a comprehensive comparison of these methods, including their accuracy in identifying cells in the hybrid E/M phenotype(s), is lacking. Here, we compare three distinct metrics that score EMT on a continuum, based on the transcriptomics signature of individual samples. Our results demonstrate that these methods exhibit good concordance among themselves in quantifying the extent of EMT in a given sample. Moreover, scoring EMT using any of the three methods discerned that cells can undergo varying extents of EMT across tumor types. Separately, our analysis also identified tumor types with maximum variability in terms of EMT and associated an enrichment of hybrid E/M signatures in these samples. Moreover, we also found that the multinomial logistic regression (MLR)-based metric was capable of distinguishing between “pure” individual hybrid E/M vs. mixtures of E and M cells. Our results, thus, suggest that while any of the three methods can indicate a generic trend in the EMT status of a given cell, the MLR method has two additional advantages: (a) it uses a small number of predictors to calculate the EMT score and (b) it can predict from the transcriptomic signature of a population whether it is comprised of “pure” hybrid E/M cells at the single-cell level or is instead an ensemble of E and M cell subpopulations.

## Introduction

The epithelial–mesenchymal transition (EMT) is a cell biological process crucial for various aspects of tumor aggressiveness – cancer metastasis (Jolly et al., 2017), resistance against cell death (Huang et al., 2013), metabolic reprogramming (Thomson et al., 2019), refractory response to chemotherapy and radiotherapy (Kurrey et al., 2009), tumor-initiation potential (Jolly et al., 2014), and immune evasion (Tripathi et al., 2016; Terry et al., 2017) – thus eventually affecting patient survival (Tan et al., 2014). EMT is a multidimensional, non-linear process that involves changes in a compendium of molecular and morphological traits, such as altered cell polarity, partial or complete loss of cell–cell adhesion, and increased migration and invasion. Cells may take different routes in this multidimensional landscape as effectively captured by recent high-throughput dynamic approaches (Karacosta et al., 2019; Watanabe et al., 2019). The trajectories taken by cancer cells in the EMT landscape may depend on the dosage and duration of the EMT induction signal (Stylianou et al., 2018; Katsuno et al., 2019; Tripathi et al., 2020), and thus may be associated with varying metastatic potency (Aiello et al., 2018) and varying degrees of resistance against different drugs (Biddle et al., 2016), thereby driving a context-specific association of patient survival with EMT (Chikaishi et al., 2011; Tan et al., 2014; Yan et al., 2016).

Initially thought of as binary, EMT is now considered as a complex process involving one or more hybrid epithelial/mesenchymal (E/M) states (Jolly and Celia-Terrassa, 2019). These hybrid E/M states can be more plastic and tumorigenic than “purely E” or “purely M” ones, thus constituting the “fittest” phenotype for metastasis (Grosse-Wilde et al., 2015; Bierie et al., 2017; Pastushenko et al., 2018; Kröger et al., 2019; Tripathi et al., 2020). Consequently, the presence and frequency of such hybrid E/M cells in primary tumors and in circulating tumor cells (CTCs) can be associated with poor patient survival (Jolly et al., 2019a; Saxena et al., 2019). Computational methods aimed at quantifying EMT on a continuous spectrum in order to enhance diagnostic, prognostic, and therapeutic intervention are therefore indispensable.

Various methods have been developed to obtain a quantitative measure of the extent of EMT (hereafter, referred to as EMT score) that cells in a given sample have undergone. Here we focus on methods accomplishing this task using the gene expression data. First, a 76-gene EMT signature (76GS; hereafter referred to as the 76GS method) was developed and validated using gene expression from non-small cell lung cancer (NSCLC) cell lines and patients treated in the BATTLE trial (Byers et al., 2013). This scoring method calculates EMT scores based on a weighted sum of the expression levels of 76 genes; the weight factor of a gene is the correlation coefficient between the expression level of that gene and that of CDH1 (E-cadherin) in that dataset; thus, the absolute EMT scores of E samples using the 76 GS method are relatively higher than those of M samples (Guo et al., 2019). Second, an EMT score separately for cell lines and tumors was developed based on a two-sample Kolmogorov–Smirnov test (KS; hereafter referred to as the KS method). This score varies on a scale of −1 to 1, with the higher scores corresponding to more M samples (Tan et al., 2014). Third, a multinomial logistic regression (MLR; hereafter referred to as the MLR method)-based model quantified the extent of EMT in a given sample on a scale of 0–2. This method particularly focuses on characterizing a hybrid E/M phenotype using the expression levels of 23 genes – 3 predictors and 20 normalizers – identified through NCI-60 gene expression data. It consequently calculates the probability that given sample belongs to E, M, or hybrid E/M categories. An EMT score is assigned based on those probabilities; the higher the score, the more M the sample is (George et al., 2017). A comparative analysis of these methods in terms of similarities, differences, strengths, and limitations, remains to be done.

Here, we present a comprehensive evaluation of these methods – 76GS, KS, and MLR – in terms of quantifying EMT and characterizing the hybrid E/M phenotype. First, we calculate the correlations observed across different *in vitro*, *in vivo*, and patient datasets, and observe good quantitative agreement among the scores calculated using these three methods. This analysis suggests that all of them, despite using varied gene lists and methods, concur in capturing a generic trend embedded in the multi-dimensional EMT gene expression landscape. Second, we identify which cancer types are more heterogeneous than others in terms of their EMT status; intriguingly, our results show that enrichment for a hybrid E/M phenotype contributes to heterogeneity. Third, we compare the ability of these methods to distinguish between “pure” individual hybrid E/M cells vs. mixtures of E and M cells that can exhibit an EMT score similar to that of hybrid E/M samples. Our results offer proof-of-principle that the MLR method can identify these differences. Overall, our results demonstrate the consistency of these EMT scoring metrics in quantifying the spectrum of EMT. Moreover, two advantages of MLR method are highlighted – namely, the use of a small number of predictors to calculate the EMT score, and the ability to characterize difference between admixtures of E and M cells vs. truly hybrid E/M cells.

## Materials and Methods

### Software and Datasets

All computational and statistical analyses were performed using R (version 3.4.0) and Bioconductor (version 3.6). Microarray datasets were downloaded using *GEOquery* R Bioconductor package (Davis and Meltzer, 2007). TCGA datasets were obtained from the *UCSC xena tools* (Wang S. et al., 2019). CCLE and NCI60 datasets were downloaded from respective websites.

### Preprocessing of Microarray Data Sets

All microarray datasets were preprocessed to obtain the gene-wise expression for each sample from probe-wise expression matrix. To map the probes to genes, relevant platform annotation files were utilized. If there were multiple probes mapping to one gene, then the mean expression of all the mapped probes was considered for that gene.

### Calculation of EMT Scores

Epithelial–mesenchymal transition (EMT) scores were calculated for samples in a particular data set using all three methods. For a particular microarray data set, expression of respective gene signatures was given as an input to calculate EMT score using all three different methods.

#### 76GS

The EMT scores were calculated based on a 76-gene expression signature reported (Byers et al., 2013; Supplementary Table S1) and the metric mentioned based on that gene signature (Guo et al., 2019). For each sample, the score was calculated as a weighted sum of 76 gene expression levels and the scores were centered by subtracting the mean across all tumor samples so that the grand mean of the score was zero. Negative scores can be interpreted as M phenotype whereas the positive scores as E.

#### MLR

The ordinal MLR method predicts EMT status based on the order structure of categories and the principle that the hybrid E/M state falls in a region intermediary to E and M. Quantitative estimates of EMT spectrum were inferred based on the assumptions and equations mentioned (George et al., 2017; Supplementary Table S2). The samples are scored ranging from 0 (pure E) to 2 (pure M), with a score of 1 indicating a maximally hybrid phenotype. These scores are calculated based on the probability of a given sample being assigned to the E, E/M, and M phenotypes.

#### KS

The KS EMT scores were calculated as previously reported (Tan et al., 2014; Supplementary Tables S3, S4). This method compares cumulative distribution functions (CDFs) of E and M signatures. First, the distance between E and M signatures was calculated via the maximum distance between their CDFs as follows: For CDFs *F*_E_(*x*) and *F*_M_(*x*) representing the levels of transcript *x* for E and M signatures, respectively, the distance between signatures is assessed by using the uniform norm

(1) $$||F_{\text{E}}-F_{\text{M}}||\equiv \max_{x}\left|F_{\text{E}}\,\left(x\right)-F_{\text{M}}\,\left(x\right)\right|$$

This quantity represents the test statistic in the subsequent two-sample test used to calculate the EMT score. The score is determined by hypothesis testing of two alternative hypotheses as follows (with the null hypothesis being that there is no difference in CDFs of M and E signatures): (1) CDF of M signature is greater than CDF of E signature. (2) CDF of E is greater than CDF of M signature. Sample with a positive EMT score is M whereas negative EMT score is associated with E phenotype.

### Correlation Analysis

Correlation between EMT scores was calculated by Pearson’s correlation, unless otherwise mentioned.

### Survival Analysis

All samples were segregated into 76GS^*high*^ and 76GS^*low*^, MLR^*high*^ and MLR^*low*^, KS^*high*^ and KS^*low*^ groups based on the mean values of respective EMT score. Observed survival distributions are graphically depicted for each method with the above-mentioned two categories.

### Mixture Curve Analysis

For each dataset analyzed using mixture curves, the most M (pure-M) and most E (pure-E) samples were identified by ordering samples based on MLR EMT score and selecting the top and bottom 35 samples, respectively. The mean or median was calculated for the pure-E and pure-M samples as a representative of the purified E or M state in the MLR predictor space. From this, the mixture curve is derived by taking all convex combinations of purified states. Individual samples within a given dataset were ranked based on their proximity to the mixture curve using the usual l_2_-norm distance. The top 10, 20, 50, and 100 samples closest to, and furthest from, the mixture curve were used as representative mixtures of E and M populations and hybrid E/M signatures, respectively.

## Results

### Concordance in Capturing EMT Response

**FIGURE 1:**
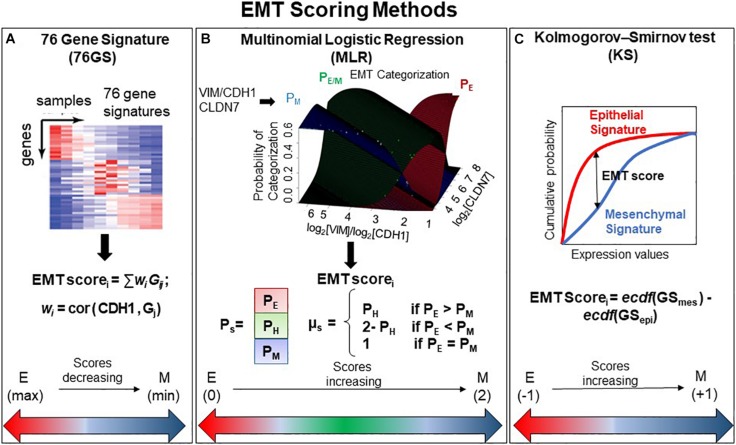
General outline of all three EMT scoring methods. **(A)** 76GS score is calculated by weighted sum of 76 genes, where EMT score*_*i*_* is score for *i*th sample, *w*_*j*_ is correlation of *j*th gene (*G*_*j*_) with CDH1 gene in that dataset to which the sample *i* belongs, *G*_*ij*_ is the *j*th gene’s normalized expression in *i*th sample. **(B)** MLR utilizes log_2_(VIM)/log_2_(CDH1) and log_2_(CLDN7) space to predict categorization of a sample into E, E/M, or M category. Where *P*_*E*_, *P*_*H*_, and *P*_*M*_ are the probabilities of a sample falling into each phenotype. EMT score*_*i*_* is the score for *i*th sample, which is defined in relation to *P*_*E*_, *P*_*H*_, and *P*_*M*_. Figure adapted from George et al. (2017) with permission. **(C)** KS score is estimated by the empirical cumulative distributions of epithelial and mesenchymal gene set, denoted by ecdf (GS_*mes*_) and ecdf (GS_*epi*_), respectively. EMT score*_*i*_* is the maximum vertical distance between the ecdf (GS_*mes*_) and ecdf (GS_*epi*_) (given by Eq. 1 in the section “Materials and Methods”) for a given sample *i*.

We used three different EMT scoring methods to quantify the extent of EMT in given transcriptomics data; each method utilizes a distinct gene set as well as a different underlying algorithm. In the 76GS method, the higher the score, the more E a sample is, given that the method calculates as weighted sum of expression levels of 76 genes, with the weight factor being correlation coefficient with levels of the canonical E marker CDH1 (Figure 1A). This method has no specific pre-defined range of values, although the range of values obtained are bounded by the maximal possible value of gene expression detected by microarray. Unlike the 76GS method, the MLR and KS methods have predefined scales for EMT scores. MLR and KS score EMT on a spectrum of [0, 2] and [−1, 1] respectively, with higher scores indicating M signatures (Figures 1B,C). While MLR and KS methods are absolute, requiring a fixed transcript signature for EMT score calculation, the 76GS method of EMT scoring depends on the number and nature of samples analyzed in a given dataset. Consequently, a hybrid E/M sample may have a (pseudo) low 76GS score whenever the available dataset contains more M samples, or a (pseudo) high score for datasets enriched in E samples. Each scoring method also varies in the number of required gene transcripts: while the MLR method utilizes 23 entries, the 76GS method requires 76 entires. The KS method utilizes 315 and 218 transcripts for tumor samples and cell lines samples, respectively.

**FIGURE 2:**
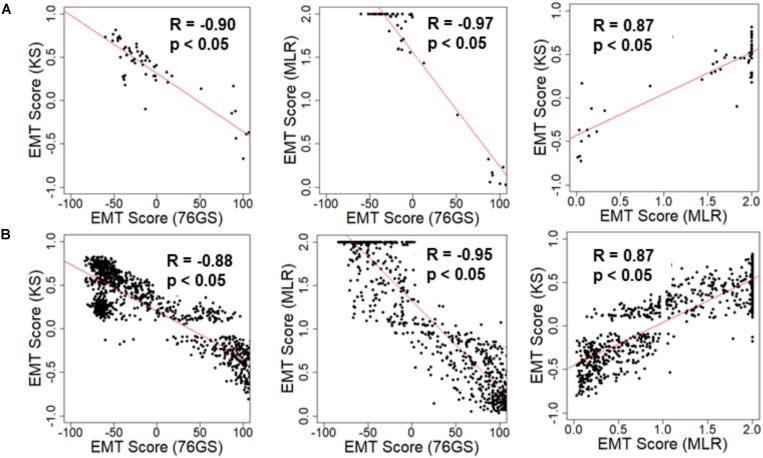
Scatter plot depicting the correlation between the EMT scores of cancer cell line samples calculated via three methods. Each pairwise relation is estimated by a linear regression line (red), Pearson’s correlation coefficient (*R*), and *p*-value (*p*) reported in each plot. **(A)** NCI60 dataset and **(B)** CCLE dataset.

We first investigated the extent of concordance in EMT scores calculated via these three methods for well-studied cohorts of cancer cell lines: NCI-60 and CCLE (Shankavaram et al., 2009; Barretina et al., 2012). We expected to see a negative correlation between EMT scores calculated via 76GS and KS methods and that between EMT scores using 76GS and MLR methods, whereas a positive correlation should exist between EMT scores from the MLR and KS methods. Indeed, for both NCI-60 and CCLE datasets, the EMT scores calculated via different methods were found to be correlated significantly with a high absolute value of correlation coefficients in the expected direction, when compared pairwise (Figure 2 and Supplementary Figure S1). Given that the three scoring methods utilize very different metrics and varying number of genes to define and quantify EMT, it was remarkable that all three showed such high consistency in scoring EMT for these datasets that contained cell lines across various cancer types.

**FIGURE 3:**
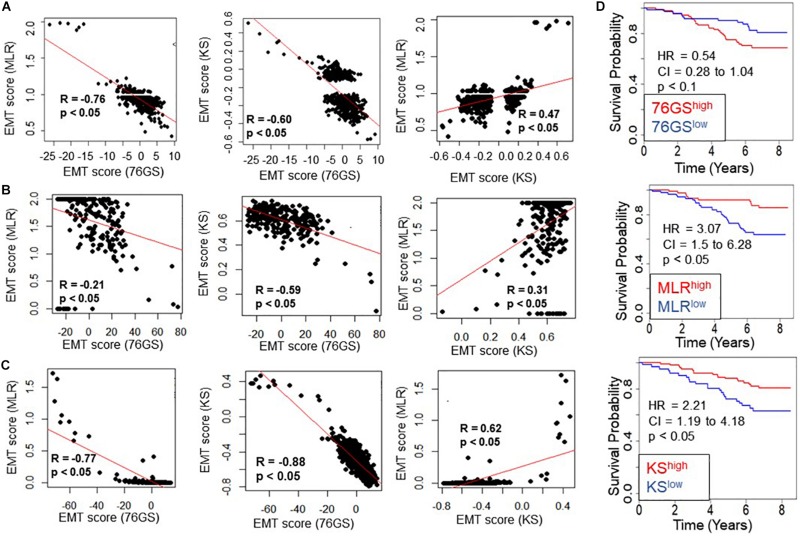
Concordance across all three EMT scoring methods in quantification of EMT and survival prediction of tumor patients. Each pairwise relation is estimated by linear regression (red), Pearson’s correlation coefficient (*R*), and *p*-value (*p*), reported in each plot. **(A)** TCGA ovarian cancer dataset, **(B)** TCGA sarcoma dataset, **(C)** TCGA breast cancer dataset. **(D)** Correlation between EMT score (high vs. low) and overall survival (OS) in breast cancer patients. Kaplan–Meier survival analysis is performed to estimate differences in survival of 76GS^*high*^, MLR^*high*^, KS^*high*^ and 76GS^*low*^, MLR^*low*^, KS^*low*^ groups, respectively, in GSE1456. *p*-values (*p*) reported are based on the log rank test. HR (hazard ratio) and confidence interval (95% CI) reported are estimated using cox regression.

Next, we investigated whether this trend was also present in the TCGA patient samples of different tumor types. Again, the trend remained consistent across tumor types – a strongly positive significant correlation between scores via MLR and KS, and a strongly negative significant correlation between scores via 76GS and KS and those via 76GS and MLR methods (Figures 3A–C and Supplementary Figure S2). Among all tumor types in TCGA data, breast cancer exhibited the highest observed correlation coefficient across methods (Figure 3C). Thus, the association between EMT scores and patient survival was assessed using breast cancer patient samples. The samples were scored using all three methods and segregated into high and low groups based on the mean value of each EMT score. The 76GS^*low*^ subgroup can be thought of as similar to the MLR^*high*^ and/or KS^*high*^ ones, given their relatively strong M signature. The three EMT scoring methods showed consistent trends in predicting overall survival highlighting that patients with a strongly M phenotype had better survival probability (Figure 3D), endorsing the emerging notion that the predominance of EMT in primary tumors and/or CTCs need not always be correlated with worse patient survival (Tan et al., 2014; Saxena et al., 2019).

Epithelial–mesenchymal transition can be driven by diverse biomechanical and/or biochemical stimuli in tumor microenvironments. TGFβ is one of the best-studied drivers of EMT, and a recent study identified a signature specific to TGFβ-induced EMT (Foroutan et al., 2017). EMT scores calculated via any of the three methods – KS, MLR, and 76GS – correlated well with the scores calculated for TGFβ-induced EMT gene signature (Supplementary Figure S3), further endorsing the equivalence of these methods in identifying the onset of EMT.

**FIGURE 4:**
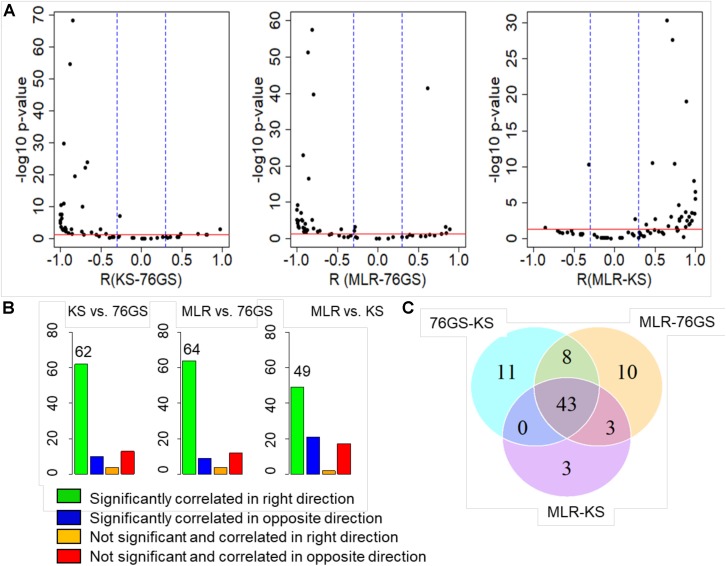
Plots depicting pairwise comparisons of all three EMT scores. **(A)** Volcano plots showing the correlation of different EMT scoring methods across 85 different GEO microarray datasets along with the *p*-values for the respective correlation coefficient values. In each case, –log10(*p*-value) is plotted as a function of Pearson’s correlation coefficient. Thresholds for correlation (*R* < –0.3 or *R* > 0.3; vertical blue lines) and *p*-values (*p* < 0.05; horizontal red line) are denoted. **(B)** Bar plots for different categories based on the correlation sign and statistical significance of all three pairwise comparisons across 85 datasets. *p* < 0.05 and *R* < −0.3 or *R* > 0.3. **(C)** Venn diagram showing the common GEO datasets across all pairwise comparisons that are significantly correlated in the expected direction.

After establishing this consistency using *in vitro* cell line datasets and TCGA patient samples, we focused on several publicly available microarray datasets including those of EMT induction or reversal, isolation of subpopulations, etc. Each dataset comprised a variety of samples in terms of different cell lines, conditions, and treatments. An analysis of different GEO datasets showed that EMT scores calculated via these three methods, when compared pairwise, were significantly correlated in the expected direction (Figure 4A and Supplementary Table S5). Out of 85 different datasets, a large percentage of them showed trends in the expected direction (62/85 in KS vs. 76GS; 64/85 in MLR vs. 76GS; 49/85 in MLR vs. KS) (Figure 4B). Strikingly, 43 datasets were found to be common across all three pairwise comparisons (Figure 4C), establishing a high degree of concordance among EMT scores calculated via these three EMT scoring methods.

**FIGURE 5:**
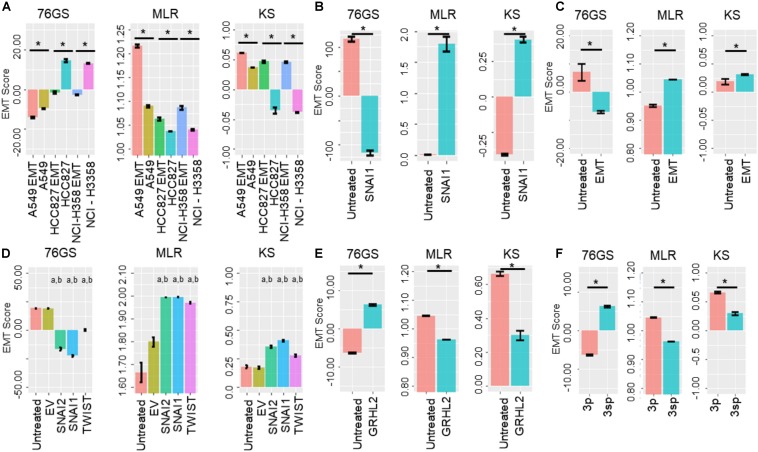
Bar plots showing EMT scores of different cell lines calculated using the three EMT scoring methods. **(A)** EMT induction is shown in three cell lines – A549, HCC87, and NCIH358 (GSE49664). **(B)** EMT induction in MCF7 cell line (GSE58252). **(C)** EMT induction in EpRas cells (GSE59922). **(D)** EMT induction by different EMT-inducing transcription factors. “a” denotes statistical significant difference (*p* < 0.05, *n* = 3, two-tailed Student’s *t*-test) for pairwise comparison of a given set with untreated (first column), “b” denotes the same when compared with empty vector (EV; second column) (GSE43495). **(E)** MET induction by GRHL2 in MDA-MB-231 cell line (GSE36081). **(F)** Two cell lines of hepatocellular carcinoma with varying EMT status (GSE26391). Each control case has been compared to EMT/MET induced case (*p < 0.05, *n* = 3, two-tailed Student’s *t*-test; error bars represent standard deviation).

Next, we investigated specific cases where EMT/MET was induced in various cell lines by different EMT/MET regulators. Lung cancer cell lines A549, HCC827, and H358 in which EMT was induced by TGFβ showed higher EMT scores using MLR and KS methods, but lower scores via 76GS method, compared to untreated ones (Figure 5A). Similarly, the E breast cancer cell line MCF-7 transfected to overexpress EMT-inducing transcription factor Snail exhibited a more M phenotype relative to the control, as identified via all three scoring methods (Figure 5B). Consistent trends were seen in EpRAS tumor cells treated with TGFβ (Figure 5C), and in human mammary E cells HMLE overexpressing one of the three EMT-inducing transcription factors (EMT-TFs) – SNAI1 (Snail), SNAI2 (Slug), and TWIST (Figure 5D). Interestingly, all three scoring methods suggested that EMT induced by Snail or Slug was stronger than that induced by Twist (Figure 5D). Further, inducing EMT via overexpression of EMT-TFs Twist, Snail, Goosecoid, or treatment with TGFβ or knockdown of E-cadherin was capable of altering the EMT scores of HMLE cells (Supplementary Figure S4A).

Additionally, these three methods also captured the reversal of EMT – M–E transition (MET) – induced by MET-inducing transcription factor GRHL2 in MDA-MB-231 cells (Figure 5E). Moreover, baseline differences in EMT status between two hepatocellular carcinoma cell lines identified experimentally (Van Zijl et al., 2011) were also recapitulated by all three scoring methods; while HCC-1.2 (referred to as 3p) showed more E features, HCC1.1 (referred to as 3sp) was relatively more M (Figure 5F). We also calculated the EMT scores for the dynamic EMT time series datasets (i.e., cases where more than two time points were available for EMT induction); all three methods were able to recapitulate the relevant trends in EMT scores as expected when EMT was induced in A549 and LNCAP cells (Supplementary Figures S4B,D). Further, all three EMT scoring methods captured the trend in the change of EMT status in prostate cancer E PC3 cells (PC3-Epi) and M cell lines derived from PC3 (PC-EMT) through interactions with macrophages (Roca et al., 2013). PC3-EMT cells transfected with ZEB1-shRNA vector (sh4), but not with the scrambled control (Scr), indicated an MET (Supplementary Figure S4C). Finally, we calculated EMT scores for a population of CTCs collected from breast cancer patients and *ex vivo* cancer models and observed heterogeneity in CTCs along the E-hybrid–M spectrum (Supplementary Figures S4E,F), reminiscent of similar observations based on immunohistochemical staining of a few canonical markers (Yu et al., 2013).

### Variability in EMT Scores Measures Tumor Heterogeneity

**FIGURE 6:**
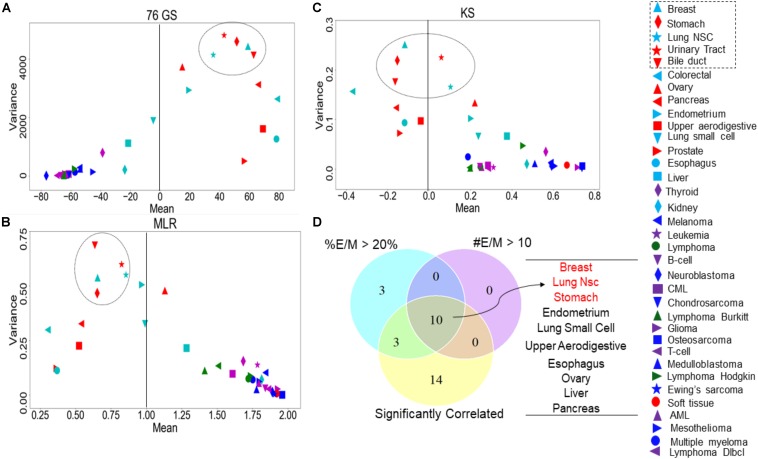
Variance and mean of EMT scores in CCLE samples grouped by tumor subtype, highlighting the most variable tumor types (circled). **(A)** 76GS EMT scores, **(B)** MLR EMT scores, and **(C)** KS EMT scores. **(D)** Venn diagram showing the overlap between each tumor type based on the abundance of hybrid samples as defined by the MLR method, where #EM > 10 denote the cases where the absolute number of hybrid E/M samples in a tumor subtype is >10; %EM > 20 denote the cases where the percentage of cell lines identified as hybrid E/M in a given tumor subtype is >20%.

Recent studies have emphasized that intra-tumor heterogeneity and inter-tumor heterogeneity can accelerate progression and metastasis (Lawson et al., 2018). Thus, we were interested in identifying which tumor types are more heterogeneous with regard to EMT scores calculated via the three methods. We grouped the CCLE samples by different tumor types and calculated the mean and variance of all EMT scores across a given tumor. The EMT scores, calculated across the three methods, showed less variation in the EMT scores of the tumor types of M origin such as sarcoma and lymphoma, compared to that of the other tumor types such as breast cancer and lung cancer (Figures 6A–C and Supplementary Table S7). The most heterogeneous tumor types identified based on the variance in EMT scores largely overlapped for all methods: (a) breast cancer, (b) stomach cancer, (c) NSCLC, (d) bile duct cancer, and (e) urinary tract cancer (Figures 6A–C). We also calculated pairwise correlations of EMT scores across all the tumor types and observed consistently significant trends (Supplementary Table S8).

One of the proposed mechanisms underlying such heterogeneity in EMT status has been E–M plasticity, i.e., the proclivity of individual cells in a population to obtain and switch among multiple phenotypic states. Such plasticity is typically seen to be higher in cells in one or more hybrid E/M states (Pastushenko and Blanpain, 2019; Tripathi et al., 2019, 2020). Thus, we asked whether the frequency of hybrid E/M phenotype contributes to heterogeneity in terms of EMT scoring. One of the EMT scoring methods – MLR – calculates the probability of a given transcriptomic profile being associated with the E, hybrid E/M, or M state, thus enabling us to identify hybrid E/M samples specifically. First, we found that the variance of EMT scores was the highest in samples identified as hybrid E/M as compared to E and M samples (Supplementary Table S6A). Consistently, a high correlation coefficient value in EMT scores was maintained, when calculated separately for CCLE samples in E, E/M, and M categories (Supplementary Table S6B). Next, we checked the relative frequency and absolute number of hybrid E/M samples (as defined by MLR method) across tumor types, among the cases where EMT scores calculated via all three methods were significantly correlated. Indeed, the tumor types that met the three conditions – (a) total number of hybrid E/M samples being more than 10, (b) percentage of hybrid E/M samples being >20%, and (c) a good correlation among all three methods – were enriched in the most variable tumor types (Figure 6D), suggesting hybrid E/M phenotypes contribute maximally to E–M heterogeneity (Supplementary Table S9).

We also calculated the correlations in EMT scores obtained from each method, after segregating the cell line samples into E, E/M, and M, based on predictions from the MLR method. The correlation coefficients within the E, E/M, and M subgroups of a given tumor subtype were observed to be somewhat different than those found for all tumor subtype samples without any partitioning into E, E/M, and M subgroups (Supplementary Table S8). These results suggest that while a generic trend in terms of EMT scores is seen across the three methods, the categorization in terms of E, E/M, and M may vary to some degree based on the EMT scoring method used. It should be noted that while the MLR method classifies samples into three broad categories (E, E/M, and M), it makes no assumption on the existence, the number, or the stability of sub-states within each category. In fact, the scores calculated using the MLR method use a continuous scale for EMT quantification, which measures the extent of EMT and thus, reflects, in principle, an entire range of different partial states of EMT.

### Individual Hybrid E/M Samples Are Different From Hybrid Mixtures of E and M

A given transcriptomic profile may be classified as hybrid E/M for several reasons: (a) the sample contains individually hybrid E/M cells (hybrids), (b) the sample contains a mixture of E and M cells (mixtures), or (c) the sample contains a combination of hybrids and mixtures. We sought to distinguish true hybrids from mixtures based on an additional feature of MLR scoring – mixture curve analysis (Jia et al., 2019). This analysis quantifies the distance of a given sample from a “mixture curve” which connects the position of mean signatures of “pure” E and “pure” M samples. The farther a given sample is from the mixture curve, the higher the likelihood of that particular sample containing truly hybrid E/M cells.

**FIGURE 7:**
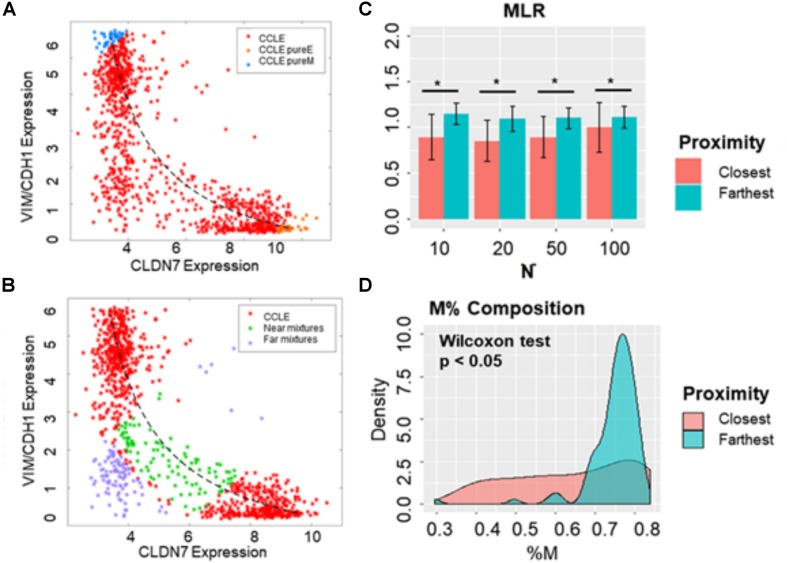
Distinguishing between hybrid E/M cells vs. mixtures of E and M cells. **(A)** Scatter plot showing CCLE cell lines that display a hybrid E/M phenotype (red) on the mixture curve (dotted curve) determined by the mean of 35 pure E (orange) and pure M (blue) reference samples in CCLE dataset. **(B)** Scatter plot showing the 100 farthest (purple) and 100 closest (green) samples based on the distance from the mixture curve. **(C)** Bar plots showing EMT scores of N (10, 20, 50, and 100) closest and farthest hybrid E/M samples from mixture curve. **(D)** Mesenchymal proportion (%M) distribution of the 100 closest and farthest hybrid samples from mixture curve. *p < 0.05, *N* = 10, 20, 50 and 100, two-tailed Student’s *t*-test; error bars represent standard deviation.

First, we determined the mixture curves based on the CCLE samples. We ranked all cell lines in the CCLE dataset based on their EMT scores and identified the top 35 most E (i.e., lowest 35 in terms of MLR EMT scores) and top 35 most M samples (i.e., highest 35 in terms of EMT MLR scores). Then, the mixture curve was determined based on the convex combinations of mean signatures of these 35 “pure” E and 35 “pure” M reference samples. All the CCLE cell lines identified as hybrid E/M were then plotted alongside the mixture curve (Figure 7A) and their distances from the curve were calculated. While some samples fell close to the curve, many deviated substantially (Figure 7B). We subsequently picked the farthest and the closest 10, 20, 50, and 100 samples from the mixture curve and calculated their EMT scores. Intriguingly, the mean EMT score of samples farthest from the mixture curve was different than that of the closest samples as calculated using MLR, irrespective of the number of samples chosen (Figure 7C). Similarly, another “mixture curve” based on median of 35 “pure” E and “pure” M reference samples was obtained from CCLE dataset (Supplementary Figure S5A); the cell lines closest to either mixture curve tended to be more E than the ones farthest from the curve (Figure 7C and Supplementary Figure S5B). Qualitatively, speaking 76GS and KS methods showed similar results (Supplementary Figures S5C–F).

In order to distinguish the hybrid E/M samples from mixtures of pure E and pure M samples, we lastly characterized the composition of the closest and farthest hybrid E/M samples by estimating the percentage of M phenotype (%M) in each sample based on the convex combination “mixture curve” in the two-dimensional space (VIM/CDH1 expression; CLDN7 expression). While the difference in mean values of the composition (%M) of closest and farthest samples was marginal, but their overall distributions in terms of %M differed substantially (Figure 7D). This analysis demonstrates the possibility of a quantifiable compositional difference between truly hybrid E/M samples and mixtures of E and M cells.

## Discussion

Epithelial–mesenchymal transition is a reversible and dynamic process which has been shown to be activated during cancer progression. EMT involves a multitude of changes at both molecular and morphological levels. Various attempts to characterize the spectrum of EMT at molecular and/or morphological levels have been made recently, enabled by latest developments in multiplex imaging, single-cell RNA-seq and inducible systems (Mandal et al., 2016; Pastushenko et al., 2018; Stylianou et al., 2018; Cook and Vanderhyden, 2019; Devaraj and Bose, 2019; Karacosta et al., 2019; Wang W. et al., 2019; Watanabe et al., 2019; Lam et al., 2020). These approaches have highlighted the dynamical nature of EMT in driving cancer progression in patients (Jolly and Celia-Terrassa, 2019), and the heterogeneity in EMT status in cell lines and patient samples (Panchy et al., 2020; Shen et al., 2020). Further, various approaches to quantify the EMT spectrum of samples based on different signatures of tumor types have been made (Foroutan et al., 2017; Puram et al., 2017). Among all the methods available for EMT scoring, we have compared the ones that are more generalized – KS (Tan et al., 2014), MLR (George et al., 2017), and 76 GS (Byers et al., 2013; Guo et al., 2019). These three methods use different combinations of genes and metrics; however, they show a very good concordance among them in terms of identifying an empirical trend along the EMT axis.

Here, we compared the aforementioned EMT scoring metrics for their ability to identify the onset and extent of EMT/MET via calculating EMT scores for cell line cohorts NCI-60 and CCLE, TCGA cohorts from multiple subtypes, and datasets containing samples with overexpression and/or knockdown of many EMT/MET inducers such as TGFβ, Snail, Slug, Twist, E-cadherin, and GRHL2 (De Craene and Berx, 2013). The remarkable concordance among EMT scores calculated via the methods analyzed above suggests the existence of a macroscopic signal that can resolve the extent of EMT in a given sample amidst the complexity of EMT and the networks regulating it. It is plausible that within these regulatory networks, there exist key nodes forming one (or more) core circuit(s) which receive(s) a large number of inputs and may have diverse outputs, reminiscent of bow-tie structures seen in biological networks of cell-fate decision-making (Friedlander et al., 2015). This idea of core circuit(s) driving EMT is substantiated by transcriptomic meta-analysis identifying common signatures for EMT driven by distinct inducers (Taube et al., 2010; Liang et al., 2016). For instance, one network motif commonly found in core circuits regulating EMT and associated traits is a mutually inhibitory feedback loop between two “master regulators” driving opposing cell phenotypes (Hong et al., 2015; Huang et al., 2015; Saha et al., 2018); for instance, ZEB1 driving EMT and miR-200 driving MET (Jia et al., 2017). An intricate coupling among such feedback loops may give rise to a spectrum of EMT phenotypes as has been seen across cancer types in cell lines, CTCs, and primary tumor biopsies (Armstrong et al., 2011; Huang et al., 2013; Schliekelman et al., 2015; Andriani et al., 2016; Iyer et al., 2019; Markiewicz et al., 2019; Varankar et al., 2019).

In addition to EMT score concordance, the three methods showed excellent agreement in their ability to identify the most EMT-variable tumors. Most tumors of M lineage, including sarcoma samples, were shown to be least variable, as evidenced by the similarity among samples having M assignment in the CCLE dataset. This contrasts with breast cancer, NSCLC, bile duct cancer, urinary tract cancer, and stomach cancer, which exhibited the largest degree of variability in terms of their inherent EMT status in addition to being less M on average. The observations concerning the EMT status of sarcomas, breast cancer, and NSCLC are well-supported by existing experimental data (Blick et al., 2008; Schliekelman et al., 2015; Jolly et al., 2019b); however, the relationship between EMT status and heterogeneity among samples of a particular tumor type requires further investigation. Our results also demonstrate a link between the predominance of hybrid E/M status and heterogeneity patterns, possibly emerging due to relatively higher plasticity of cells in one or more hybrid E/M phenotypes (Pastushenko et al., 2018; Tripathi et al., 2020). Our findings are clinically relevant as tumor types having a greater number of hybrid E/M cells may require alternative treatment strategies compared to those containing predominantly E or predominantly M populations, necessitating future investigations into improved therapeutic design based on an analysis of EMT status and variability.

This comparative analysis of the three methods shows two key advantages of MLR method. First, it uses the least number of genes to calculate an EMT score – 23 genes required by MLR compared to 76 genes by 76GS, and 315 genes for tumor and 218 genes for cell lines by KS. This feature is important because 23 genes can be relatively easily measured experimentally without microarray or RNA-seq. Second, the MLR method, by virtue of its underlying theoretical framework, is capable of isolating hybrid E/M samples and has been expanded to identify whether the resultant gene expression is more likely to derive from “true” individual hybrid E/M samples or admixtures of E and M samples. While, in theory, other methods could adopt similar adaptations to address this issue in the future, the resolution of E, M, and hybrid E/M populations through those methods would require analyzing a higher dimensional subspace of the original predictors, given the large number of genes used by those methods to calculate EMT scores. This feature contrasts with that of MLR method, where the mixture analysis is performed directly on the two-dimensional EMT predictor space (CLDN7 and VIM/CDH1) utilized by this method. Distinguishing between these possibilities is critical because the behavior of mixtures of E and M samples vs. truly hybrid E/M samples can be strikingly different; a recent study showed that the presence of hybrid E/M cells is essential to form tumors in mice, a task which could not be achieved as efficiently by co-cultures of E and M cells alone (Kröger et al., 2019). Previously, multiple studies have implicated the role of hybrid E/M phenotype with worse survival (Grosse-Wilde et al., 2015; Grigore et al., 2016). To date, it has not been established whether it is pure hybrids or mixtures of E and M cells which correlate with clinically observed parameters. Our results highlight the utility of using the MLR method for effectively distinguishing between these two possibilities, and future work should address the relationship between the purity of hybrid E/M samples and clinical outcome.

Our analysis shown here suffers from following limitations. First, in terms of classifying hybrid E/M into “pure” hybrid E/M vs. mixtures of E and M subpopulations, we have considered mutually exclusive criteria: (a) a sample identified as hybrid E/M at a bulk level contains mixtures of E and M subpopulations, and (b) a sample identified as hybrid E/M at a bulk level contains all “true” hybrid E/M cells. However, many cell lines may contain cells in each of the three phenotypes in varying ratios (Ruscetti et al., 2016; George et al., 2017; Jia et al., 2019). Thus, future efforts should aim to identify the relative proportions of these three different phenotypes in a given sample. Second, although we show that among the samples identified to be lying closest vs. farthest from the “mixture curve” by MLR, all three EMT scoring metrics suggested that the ones lying closest to the curve are more E than the ones lying farthest from the same, we lack a clear biological interpretation of this observation. Future efforts will focus on comparing the morphological and functional behavior of the CCLE cell lines identified to be closest vs. farthest from the “mixture curve” generated based on the CCLE samples. Third, our current efforts focus on microarray data because the gene signatures utilized by all three methods were identified on this platform. Although the MLR method has been implemented on RNA-seq datasets by regressing the values obtained from microarray and RNA-seq analysis on a case-by-case basis (Kilinc et al., 2019; Lourenco et al., 2020), varying sensitivity of microarray and RNA-seq methods needs to be incorporated for future efforts in assessing these EMT scoring methods systematically.

## Data Availability Statement

All codes used for the analysis in this article can be accessed through the following link: https://github.com/priyanka8993/EMT_score_calculation.

## Author Contributions

MJ conceived and oversaw the research. PC, ST, and JG conducted the research. All authors analyzed the data and wrote the manuscript.

## Conflict of Interest

The authors declare that the research was conducted in the absence of any commercial or financial relationships that could be construed as a potential conflict of interest.

## Supplementary Material

FIGURE S1 Scatter plot depicting the correlation between the EMT scores of cancer cell line samples, calculated via three EMT scoring methods. Each pairwise relation is estimated by a linear regression line (red), Spearman’s correlation coefficient (*R*) and *p*-value (*p*) reported in each plot. **(A)** NCI60 dataset and **(B)** CCLE dataset.

FIGURE S2 Scatter plot depicting the correlation between the EMT scores of different tumor types in TCGA dataset, calculated via three methods. Each pairwise relation is estimated by a linear regression line (red), Pearson’s correlation coefficient (*R*), and *p*-value (*p*) reported in each plot. **(A)** Lung squamous cell cancer, **(B)** colon adenocarcinoma, and **(C)** colon and rectal adenocarcinoma.

FIGURE S3 EMT score correlation with TGFβ-specific EMT scoring method in CCLE dataset. **(A)** Pearson’s correlation coefficient and **(B)** Spearman’s correlation. Correlation coefficient (*R*) and *p*-value (*p*) reported in each plot.

FIGURE S4 EMT scores of different EMT time series datasets and CTCs. **(A)** GSE24202 – EMT induction by different EMT regulators. **(B)** GSE84002 – EMT and MET induction over time by GFP, SNAI1 and SNAI2. **(C)** GSE43489 – EMT/MET induction in PC3 cell line. **(D)** GSE17708 – EMT induction over time. **(E)** GSE55470 – CTCs from breast cancer patients. **(F)** GSE50991 – CTCs from *ex vivo* lung cancer model (^∗^*p* < 0.05, *n* = 3, two-tailed Student’s *t*-test; error bars represent standard deviation for *n* = 3). Graphs **(E)** and **(F)** represent kernel density plots.

FIGURE S5 **(A)** Scatter plot showing 100 farthest and closest samples based on the distance from mixture curve defined by median of 35 most pure E and pure M CCLE samples. **(B)** MLR EMT score for *N* (10,20,50,100) closest and farthest hybrid samples from median mixture curve. Bar plots showing EMT scores of *N* (10, 20, 50, 100) closest and farthest hybrid samples from mean mixture curve. **(C)** 76GS EMT score and **(D)** KS EMT score. Bar plots showing EMT scores of *N* (10, 20, 50, 100) closest and farthest hybrid samples from median mixture curve. **(E)** 76GS EMT score **(F)** KS EMT score (^∗^*p* < 0.05, *N* = 10, 20, 50, and 100, two-tailed Student’s *t*-test; error bars represent standard deviation for the given value of *N*).

TABLE S1 76 gene signatures.

TABLE S2 List of predictors and normalizers used for calculation of EMT using MLR method.

TABLE S3 Epithelial and mesenchymal signature used in KS-statistic (tumor signature).

TABLE S4 Epithelial and mesenchymal signature used in KS-statistic (cell line signature).

TABLE S5 EMT score correlation in the list of 85 microarray GEO datasets.

TABLE S6 EMT scores in E, E/M and M categories of CCLE samples, as defined by MLR EMT scores. **(A)** Mean and standard deviation of EMT scores in E, E/M and M samples. **(B)** Correlation between EMT scores across E, E/M and M categories.

TABLE S7 Most variable and least variable tumor types based on the coefficient of variation of EMT scores.

TABLE S8 Pairwise correlation between all three EMT scores in subcategories (E, E/M, and M) across all tumor types of CCLE data.

TABLE S9 Abundance of hybrid E/M samples in different tumor types.

## References

[B1] Aiello N. M. Maddipati R. Norgard R. J. Balli D. Li J. Yuan S. (2018). EMT subtype influences epithelial plasticity and mode of cell migration. *Dev. Cell* 45 681.e4–695.e4. 10.1016/J.DEVCEL.2018.05.027 29920274 PMC6014628

[B2] Andriani F. Bertolini G. Facchinetti F. Baldoli E. Moro M. Casalini P. (2016). Conversion to stem-cell state in response to microenvironmental cues is regulated by balance between epithelial and mesenchymal features in lung cancer cells. *Mol. Oncol.* 10 253–271. 10.1016/j.molonc.2015.10.002 26514616 PMC5528953

[B3] Armstrong A. J. Marengo M. S. Oltean S. Kemeny G. Bitting R. L. Turnbull J. D. (2011). Circulating tumor cells from patients with advanced prostate and breast cancer display both epithelial and mesenchymal markers. *Mol. Cancer Res.* 9 997–1007. 10.1158/1541-7786.MCR-10-0490 21665936 PMC3157566

[B4] Barretina J. Caponigro G. Stransky N. Venkatesan K. Margolin A. A. Kim S. (2012). The Cancer Cell Line Encyclopedia enables predictive modelling of anticancer drug sensitivity. *Nature* 483 603–607. 10.1038/nature11003 22460905 PMC3320027

[B5] Biddle A. Gammon L. Liang X. Costea D. E. Mackenzie I. C. (2016). Phenotypic plasticity determines cancer stem cell therapeutic resistance in oral squamous cell carcinoma. *EBioMedicine* 4 138–145. 10.1016/j.ebiom.2016.01.007 26981578 PMC4776071

[B6] Bierie B. Pierce S. E. Kroeger C. Stover D. G. Pattabiraman D. R. Thiru P. (2017). Integrin-β4 identifies cancer stem cell-enriched populations of partially mesenchymal carcinoma cells. *Proc. Natl. Acad. Sci. U.S.A.* 114 E2337–E2346. 10.1073/pnas.1618298114 28270621 PMC5373369

[B7] Blick T. Widodo E. Hugo H. Waltham M. Lenburg M. E. Neve R. M. (2008). Epithelial mesenchymal transition traits in human breast cancer cell lines. *Clin. Exp. Metastasis* 25 629–642. 10.1007/s10585-008-9170-6 18461285

[B8] Byers L. A. Diao L. Wang J. Saintigny P. Girard L. Peyton M. (2013). An epithelial-mesenchymal transition gene signature predicts resistance to EGFR and PI3K inhibitors and identifies Axl as a therapeutic target for overcoming EGFR inhibitor resistance. *Clin. Cancer Res.* 19 279–290. 10.1158/1078-0432.CCR-12-1558 23091115 PMC3567921

[B9] Chikaishi Y. Uramoto H. Tanaka F. (2011). The EMT status in the primary tumor does not predict postoperative recurrence or disease-free survival in lung adenocarcinoma. *Anticancer Res.* 31 4451–4456. 22199314

[B10] Cook D. P. Vanderhyden B. C. (2019). Comparing transcriptional dynamics of the epithelial-mesenchymal transition. *bioRxiv* [Preprint]. 10.1101/732412 28973892

[B11] Davis S. Meltzer P. S. (2007). GEOquery: a bridge between the Gene Expression Omnibus (GEO) and BioConductor. *Bioinformatics* 23 1846–1847. 10.1093/bioinformatics/btm254 17496320

[B12] De Craene B. Berx G. (2013). Regulatory networks defining EMT during cancer initiation and progression. *Nat. Rev. Cancer* 13 97–110. 10.1038/nrc3447 23344542

[B13] Devaraj V. Bose B. (2019). Morphological state transition dynamics in EGF-induced epithelial to mesenchymal transition. *J. Clin. Med.* 8:911. 10.3390/jcm8070911 31247884 PMC6678216

[B14] Foroutan M. Cursons J. Hediyeh-Zadeh S. Thompson E. W. Davis M. J. (2017). A Transcriptional program for detecting TGFβ-induced EMT in cancer. *Mol. Cancer Res.* 15 619–631. 28119430 10.1158/1541-7786.MCR-16-0313

[B15] Friedlander T. Mayo A. E. Tlusty T. Alon U. (2015). Evolution of bow-tie architectures in biology. *PLoS Comput. Biol.* 11:e1004055. 10.1371/journal.pcbi.1004055 25798588 PMC4370773

[B16] George J. T. Jolly M. K. Xu S. Somarelli J. A. Levine H. (2017). Survival outcomes in cancer patients predicted by a partial EMT gene expression scoring metric. *Cancer Res.* 77 6415–6428. 10.1158/0008-5472.CAN-16-3521 28947416 PMC5690883

[B17] Grigore A. Jolly M. K. Jia D. Farach-Carson M. Levine H. (2016). Tumor budding: the name is EMT. partial EMT. *J. Clin. Med.* 5:51. 10.3390/jcm5050051 27136592 PMC4882480

[B18] Grosse-Wilde A. Fouquier d’ Herouei A. McIntosh E. Ertaylan G. Skupin A. Kuestner R. E. (2015). Stemness of the hybrid epithelial/mesenchymal state in breast cancer and its association with poor survival. *PLoS One* 10:e0126522. 10.1371/journal.pone.0126522 26020648 PMC4447403

[B19] Guo C. C. Majewski T. Zhang L. Yao H. Bondaruk J. Wang Y. (2019). Dysregulation of EMT drives the progression to clinically aggressive sarcomatoid bladder cancer. *Cell Rep.* 27 1781.e4–1793.e4. 10.1016/j.celrep.2019.04.048 31067463 PMC6546434

[B20] Hong T. Watanabe K. Ta C. H. Villarreal-Ponce A. Nie Q. Dai X. (2015). An Ovol2-Zeb1 mutual inhibitory circuit governs bidirectional and multi-step transition between epithelial and mesenchymal states. *PLoS Comput. Biol.* 11:e1004569. 10.1371/journal.pcbi.1004569 26554584 PMC4640575

[B21] Huang B. Jolly M. K. Lu M. Tsarfaty I. Ben-Jacob E. Onuchic J. N. (2015). Modeling the transitions between collective and solitary migration phenotypes in cancer metastasis. *Sci. Rep.* 5:17379. 10.1038/srep17379 26627083 PMC4667179

[B22] Huang R. Y.-J. Wong M. K. Tan T. Z. Kuay K. T. Ng A. H. Chung V. Y. (2013). An EMT spectrum defines an anoikis-resistant and spheroidogenic intermediate mesenchymal state that is sensitive to e-cadherin restoration by a src-kinase inhibitor, saracatinib (AZD0530). *Cell Death Dis.* 4:e915. 10.1038/cddis.2013.442 24201814 PMC3847320

[B23] Iyer A. Gupta K. Sharma S. Hari K. Lee Y. F. Ramalingan N. (2019). Integrative analysis and machine learning based characterization of single circulating tumor cells. *bioRxiv* [Preprint]. 10.1101/867200 PMC7230872 32331451

[B24] Jia D. George J. T. Tripathi S. C. Kundnani D. L. Lu M. Hanash S. M. (2019). Testing the gene expression classification of the EMT spectrum. *Phys. Biol.* 16:025002. 10.1088/1478-3975/aaf8d4 30557866 PMC7179477

[B25] Jia D. Jolly M. K. Tripathi S. C. Den Hollander P. Huang B. Lu M. (2017). Distinguishing mechanisms underlying EMT tristability. *Cancer Converg.* 1:2. 10.1101/098962 29623961 PMC5876698

[B26] Jolly M. K. Celia-Terrassa T. (2019). Dynamics of phenotypic heterogeneity associated with EMT and stemness during cancer progression. *J. Clin. Med.* 8:1452. 10.3390/jcm8101542 31557977 PMC6832750

[B27] Jolly M. K. Huang B. Lu M. Mani S. A. Levine H. Ben-Jacob E. (2014). Towards elucidating the connection between epithelial-mesenchymal transitions and stemness. *J. R. Soc. Interface* 11:20140962. 10.1098/rsif.2014.0962 25339690 PMC4223923

[B28] Jolly M. K. Somarelli J. A. Sheth M. Biddle A. Tripathi S. C. Armstrong A. J. (2019a). Hybrid epithelial/mesenchymal phenotypes promote metastasis and therapy resistance across carcinomas. *Pharmacol. Ther.* 194 161–184. 10.1016/j.pharmthera.2018.09.007 30268772

[B29] Jolly M. K. Ware K. E. Gilja S. Somarelli J. A. Levine H. (2017). EMT and MET: necessary or permissive for metastasis? *Mol. Oncol.* 11 755–769. 10.1002/1878-0261.12083 28548345 PMC5496498

[B30] Jolly M. K. Ware K. E. Xu S. Gilja S. Shetler S. Yang Y. (2019b). E-cadherin represses anchorage-independent growth in sarcomas through both signaling and mechanical mechanisms. *Mol. Cancer Res.* 17 1391–1402. 10.1158/1541-7786.MCR-18-0763 30862685 PMC6548594

[B31] Karacosta L. G. Anchang B. Ignatiadis N. Kimmey S. C. Benson J. A. Shrager J. B. (2019). Mapping lung cancer epithelial-mesenchymal transition states and trajectories with single-cell resolution. *Nat. Commun.* 10:5587. 10.1101/570341 31811131 PMC6898514

[B32] Katsuno Y. Meyer D. S. Zhang Z. Shokat K. M. Akhurst R. J. Miyazono K. (2019). Chronic TGF-β exposure drives stabilized EMT, tumor stemness, and cancer drug resistance with vulnerability to bitopic mTOR inhibition. *Sci. Signal.* 12:eaau8544. 10.1126/scisignal.aau8544 30808819 PMC6746178

[B33] Kilinc A. N. Sugiyama N. Reddy Kalathur R. K. Antoniadis H. Birogul H. Ishay-Ronen D. (2019). Histone deacetylases, Mbd3/NuRD, and Tet2 hydroxylase are crucial regulators of epithelial–mesenchymal plasticity and tumor metastasis. *Oncogene* 39 1498–1513. 31666683 10.1038/s41388-019-1081-2

[B34] Kröger C. Afeyan A. Mraz J. Eaton E. N. Reinhardt F. Khodor Y. L. (2019). Acquisition of a hybrid E/M state is essential for tumorigenicity of basal breast cancer cells. *Proc. Natl. Acad. Sci. U.S.A.* 116 7353–7362. 10.1073/pnas.1812876116 30910979 PMC6462070

[B35] Kurrey N. K. Ghanate A. D. Chaskar P. D. Doiphode R. Y. Bapat S. A. (2009). Snail and slug mediate radioresistance and chemoresistance by antagonizing p53-mediated apoptosis and acquiring a stem-like phenotype in ovarian cancer cells. *Stem Cells* 27 2059–2068. 10.1002/stem.154 19544473

[B36] Lam V. Nguyen T. Bui V. Chung B. M. Chang L. C. Nehmetallah G. (2020). Quantitative scoring of epithelial and mesenchymal qualities of cancer cells using machine learning and quantitative phase imaging. *J. Biomed. Opt.* 25 1–17. 10.1117/1.JBO.25.2.026002 32072775 PMC7026523

[B37] Lawson D. A. Kessenbrock K. Davis R. T. Pervolarakis N. Werb Z. (2018). Tumour heterogeneity and metastasis at single-cell resolution. *Nat. Cell Biol.* 20 1349–1360. 10.1038/s41556-018-0236-7 30482943 PMC6477686

[B38] Liang L. Sun H. Zhang W. Zhang M. Yang X. Kuang R. (2016). Meta-analysis of EMT datasets reveals different types of EMT. *PLoS One* 11:e0156839. 10.1371/journal.pone.0156839 27258544 PMC4892621

[B39] Lourenco A. R. Ban Y. Crowley M. J. Lee S. B. Ramchandani D. Du W. (2020). Differential contributions of pre- and post-EMT tumor cells in breast cancer metastasis. *Cancer Res.* 80 163–169. 10.1158/0008-5472.CAN-19-1427 31704888 PMC6980649

[B40] Mandal M. Ghosh B. Anura A. Mitra P. Pathak T. Chatterjee J. (2016). Modeling continuum of epithelial mesenchymal transition plasticity. *Integr. Biol.* 8 167–176. 10.1039/C5IB00219B 26762753

[B41] Markiewicz A. Topa J. Nagel A. Skokowski J. Seroczynska B. Stokowy T. (2019). Spectrum of epithelial-mesenchymal transition phenotypes in circulating tumour cells from early breast cancer patients. *Cancers (Basel).* 11:E59. 10.3390/cancers11010059 30634453 PMC6356662

[B42] Panchy N. Azeredo-Tseng C. Luo M. Randall N. Hong T. (2020). Integrative transcriptomic analysis reveals a multiphasic epithelial–mesenchymal spectrum in cancer and non-tumorigenic cells. *Front. Oncol.* 9:1479. 10.3389/fonc.2019.01479 32038999 PMC6987415

[B43] Pastushenko I. Blanpain C. (2019). EMT transition states during tumor progression and metastasis. *Trends Cell Biol.* 29 212–226. 10.1016/j.tcb.2018.12.001 30594349

[B44] Pastushenko I. Brisebarre A. Sifrim A. Fioramonti M. Revenco T. Boumahdi S. (2018). Identification of the tumour transition states occurring during EMT. *Nature* 556 463–468. 10.1038/s41586-018-0040-3 29670281

[B45] Puram S. V. Tirosh I. Parikh A. S. Patel A. P. Yizhak K. Gillespie S. (2017). Single-cell transcriptomic analysis of primary and metastatic tumor ecosystems in head and neck cancer. *Cell* 171 1611–1624. 10.1016/j.cell.2017.10.044 29198524 PMC5878932

[B46] Roca H. Hernandez J. Weidner S. McEachin R. C. Fuller D. Sud S. (2013). Transcription factors OVOL1 and OVOL2 induce the mesenchymal to epithelial transition in human cancer. *PLoS One* 8:e76773. 10.1371/journal.pone.0076773 24124593 PMC3790720

[B47] Ruscetti M. Dadashian E. L. Guo W. Quach B. Mulholland D. J. Park J. W. (2016). HDAC inhibition impedes epithelial-mesenchymal plasticity and suppresses metastatic, castration-resistant prostate cancer. *Oncogene* 35 3781–3795. 10.1038/onc.2015.444 26640144 PMC4896852

[B48] Saha M. Kumar S. Bukhari S. Balaji S. Kumar P. Hindupur S. (2018). AMPK-Akt double-negative feedback loop in breast cancer cells regulates their adaptation to matrix deprivation. *Cancer Res.* 78 1497–1510. 10.1158/0008-5472.CAN-17-2090 29339542 PMC6033311

[B49] Saxena K. Subbalakshmi A. R. Jolly M. K. (2019). Phenotypic heterogeneity in circulating tumor cells and its prognostic value in metastasis and overall survival. *EBioMedicine* 46 4–5. 10.1016/j.ebiom.2019.07.074 31399383 PMC6712058

[B50] Schliekelman M. J. Taguchi A. Zhu J. Dai X. Rodriguez J. Celiktas M. (2015). Molecular portraits of epithelial, mesenchymal, and hybrid states in lung adenocarcinoma and their relevance to survival. *Cancer Res.* 75 1789–1800. 10.1158/0008-5472.CAN-14-2535 25744723 PMC4846295

[B51] Shankavaram U. T. Varma S. Kane D. Sunshine M. Chary K. K. Reinhold W. C. (2009). CellMiner: a relational database and query tool for the NCI-60 cancer cell lines. *BMC Genomics* 10:277. 10.1186/1471-2164-10-277 19549304 PMC2709662

[B52] Shen Y. Schmidt B. U. S. Kubitschke H. Morawetz E. W. Wolf B. Käs J. A. (2020). Detecting heterogeneity in and between breast cancer cell lines. *Cancer Converg* 4:1. 10.1186/s41236-020-0010-1 32090168 PMC6997265

[B53] Stylianou N. Lehman M. L. Wang C. Fard A. T. Rockstroh A. Fazli L. (2018). A molecular portrait of epithelial–mesenchymal plasticity in prostate cancer associated with clinical outcome. *Oncogene* 38 913–934. 30194451 10.1038/s41388-018-0488-5 PMC6514858

[B54] Tan T. Z. Miow Q. H. Miki Y. Noda T. Mori S. Huang R. Y. (2014). Epithelial-mesenchymal transition spectrum quantification and its efficacy in deciphering survival and drug responses of cancer patients. *EMBO Mol. Med.* 6 1279–1293. 10.15252/emmm.201404208 25214461 PMC4287932

[B55] Taube J. H. Herschkowitz J. I. Komurov K. Zhou A. Y. Gupta S. Yang J. (2010). Core epithelial-to-mesenchymal transition interactome gene-expression signature is associated with claudin-low and metaplastic breast cancer subtypes. *Proc. Natl. Acad. Sci. U.S.A.* 107 15449–15454. 10.1073/pnas.1004900107 20713713 PMC2932589

[B56] Terry S. Savagner P. Ortiz-Cuaran S. Mahjoubi L. Saintigny P. Thiery J. P. (2017). New insights into the role of EMT in tumor immune escape. *Mol. Oncol.* 11 824–846. 10.1002/1878-0261.12093 28614624 PMC5496499

[B57] Thomson T. M. Balcells C. Cascante M. (2019). Metabolic plasticity and epithelial-mesenchymal transition. *J. Clin. Med.* 8:967. 10.3390/jcm8070967 31277295 PMC6678349

[B58] Tripathi S. Chakraborty P. Levine H. Jolly M. K. (2020). A mechanism for epithelial-mesenchymal heterogeneity in a population of cancer cells. *PLoS Comput. Biol.* 16:e1007619. 10.1101/592691 32040502 PMC7034928

[B59] Tripathi S. Kessler D. A. Levine H. (2019). Biological regulatory networks are minimally frustrated. *arXiv* [Preprint]. Available online at: https://arxiv.org/abs/1911.10252 (accessed September 15, 2019).

[B60] Tripathi S. C. Peters H. L. Taguchi A. Katayama H. Wang H. Momin A. (2016). Immunoproteasome deficiency is a feature of non-small cell lung cancer with a mesenchymal phenotype and is associated with a poor outcome. *Proc. Natl. Acad. Sci. U.S.A.* 113 E1555–E1564. 10.1073/pnas.1521812113 26929325 PMC4801290

[B61] Van Zijl F. Mall S. Machat G. Pirker C. Zeillinger R. Weinhaeusel A. (2011). A human model of epithelial to mesenchymal transition to monitor drug efficacy in hepatocellular carcinoma progression. *Mol. Cancer Ther.* 10 850–860. 10.1158/1535-7163.MCT-10-0917 21364009

[B62] Varankar S. S. Kamble S. S. Mali A. M. More M. M. Abraham A. Kumar B. (2019). Functional balance between TCF21-Slug defines cellular plasticity and sub-classes in high-grade serous ovarian cancer. *Carcinogenesis* 10.1093/carcin/bgz119 [Epub ahead of print]. 31241128

[B63] Wang S. Zhang J. He Z. Wu K. Liu X. S. (2019). The predictive power of tumor mutational burden in lung cancer immunotherapy response is influenced by patients’ sex. *Int. J. Cancer* 145 2840–2849. 10.1002/ijc.32327 30972745

[B64] Wang W. Douglas D. Zhang J. Chen Y.-J. Cheng Y.-Y. Kumari S. (2019). M-TRACK: a platform for live cell multiplex imaging reveals cell phenotypic transition dynamics inherently missing in snapshot data. *bioRxiv* [Preprint]. 10.1101/2019.12.12.874248 PMC7473671 32917609

[B65] Watanabe K. Panchy N. Noguchi S. Suzuki H. Hong T. (2019). Combinatorial perturbation analysis reveals divergent regulations of mesenchymal genes during epithelial-to-mesenchymal transition. *npj Syst*. *Biol. Appl.* 5:21. 10.1038/s41540-019-0097-0 31275609 PMC6570767

[B66] Yan S. Holderness B. M. Li Z. Seidel G. D. Gui J. Fisher J. L. (2016). Epithelial-mesenchymal expression phenotype of primary melanoma and matched metastases and relationship with overall survival. *Anticancer Res.* 36 6449–6456. 10.21873/anticanres.11243 27919967 PMC5576452

[B67] Yu M. Bardia A. Wittner B. S. Stott S. L. Smas M. E. Ting D. T. (2013). Circulating breast tumor cells exhibit dynamic changes in epithelial and mesenchymal composition. *Science* 339 580–584. 10.1126/science.1228522 23372014 PMC3760262

