# Supplementary material for: Comparative Study of Transcriptomics-Based Scoring Metrics for the Epithelial-Hybrid-Mesenchymal Spectrum
Source: Front Bioeng Biotechnol. 2020 Mar 20;8:220. doi: 10.3389/fbioe.2020.00220 (PMC7100584; doi:10.3389/fbioe.2020.00220)
Supplement: FIGURE S1 — Scatter plot depicting the correlation between the EMT scores of cancer cell line samples, calculated via three EMT scoring methods. Each pairwise relation is estimated by a linear regression line (red), Spearman’s correlation coefficient (R) and p-value (p) reported in each plot. (A) NCI60 dataset and (B) CCLE dataset. [file Data_Sheet_1.pdf]

## Supplementary Figures

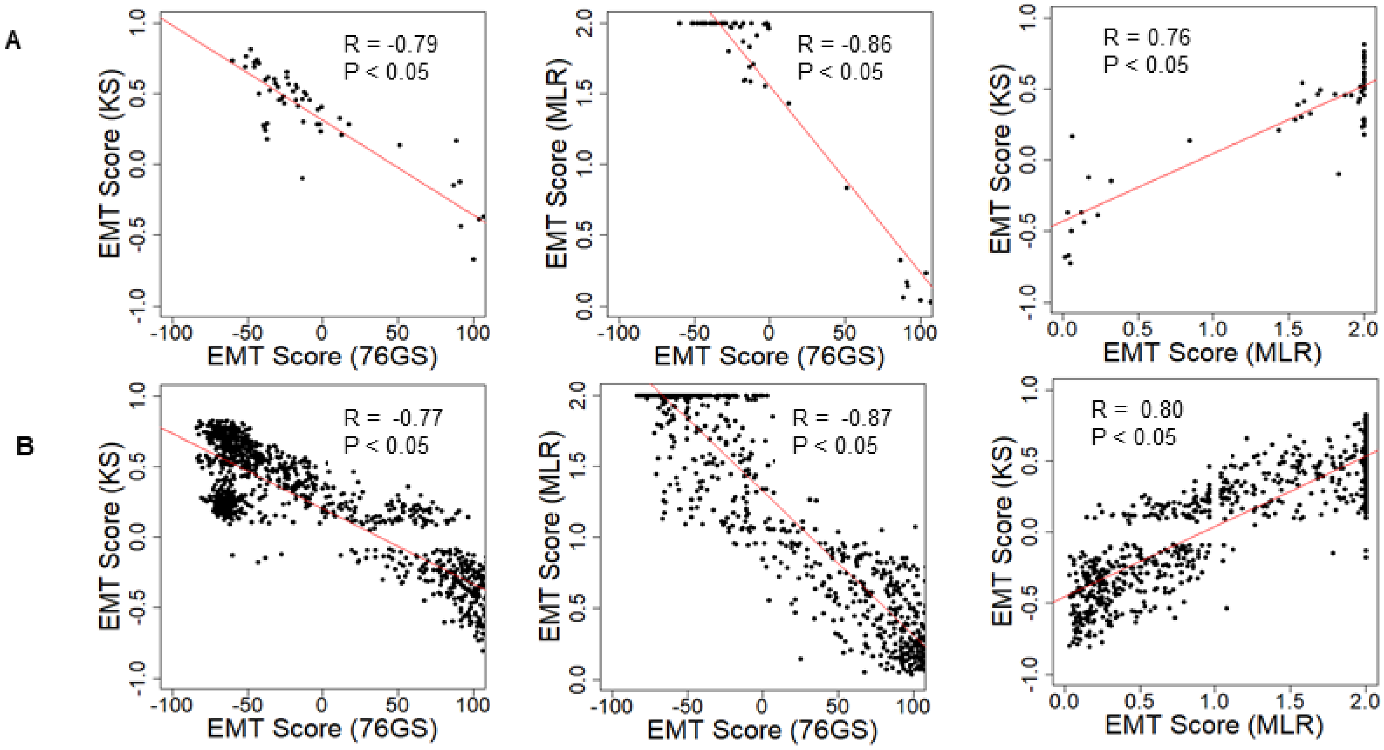


**Figure S1:** *Scatter plot depicting the correlation between the EMT scores of cancer cell line samples, calculated via three EMT scoring methods. Each pairwise relation is estimated by a linear regression line (red), Spearman's correlation coefficient (R) and p-value (p) reported in each plot.* ***A)*** *NCI60 dataset* ***B)*** *CCLE dataset*

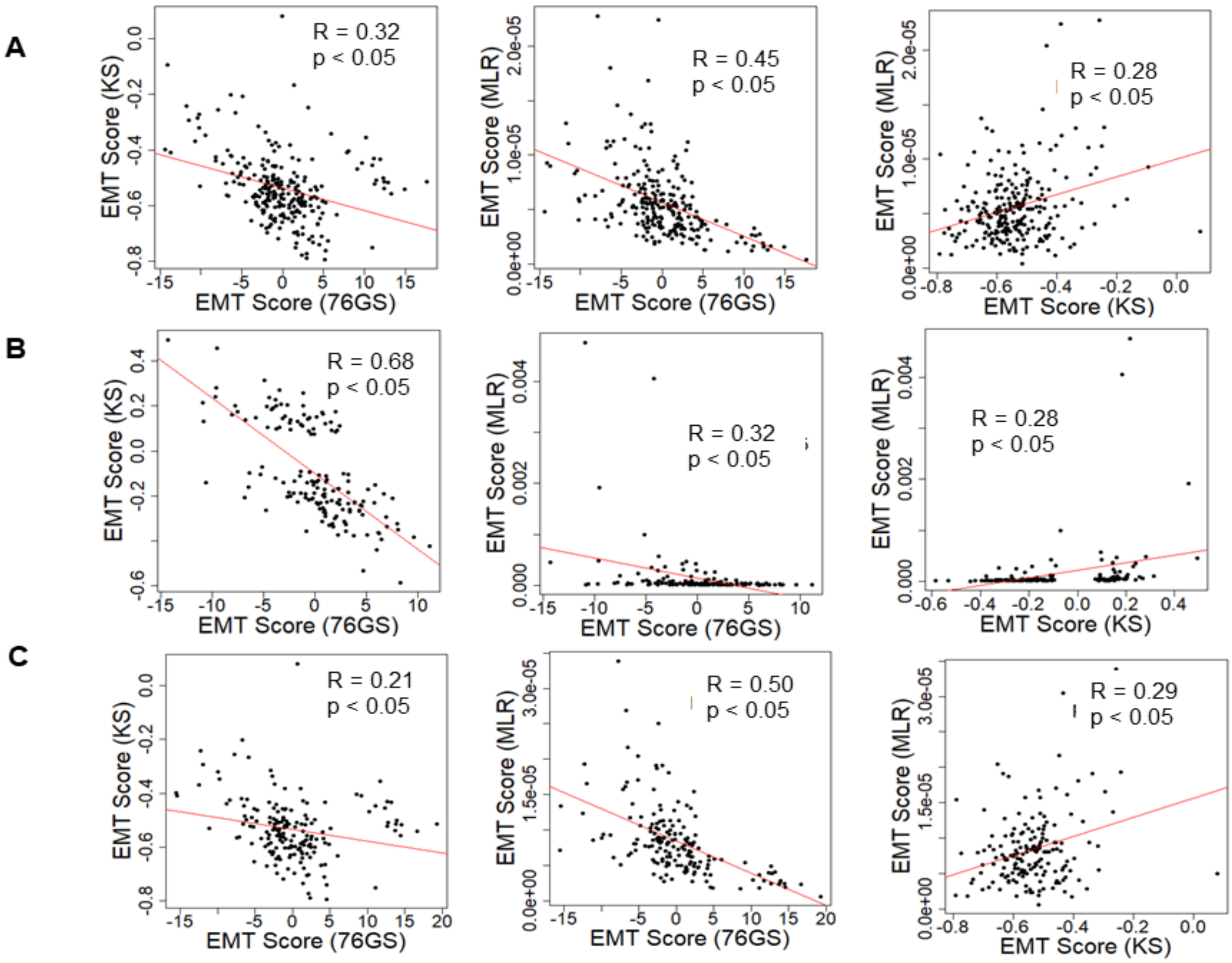


**Figure S2:** *Scatter plot depicting the correlation between the EMT scores of different tumor types in TCGA dataset, calculated via three methods. Each pairwise relation is estimated by a linear regression line (red), Pearson's correlation coefficient (R) and p-value (p) reported in each plot. (A) Lung squamous cell cancer (B) Colon adenocarcinoma (C) Colon and rectal adenocarcinoma*

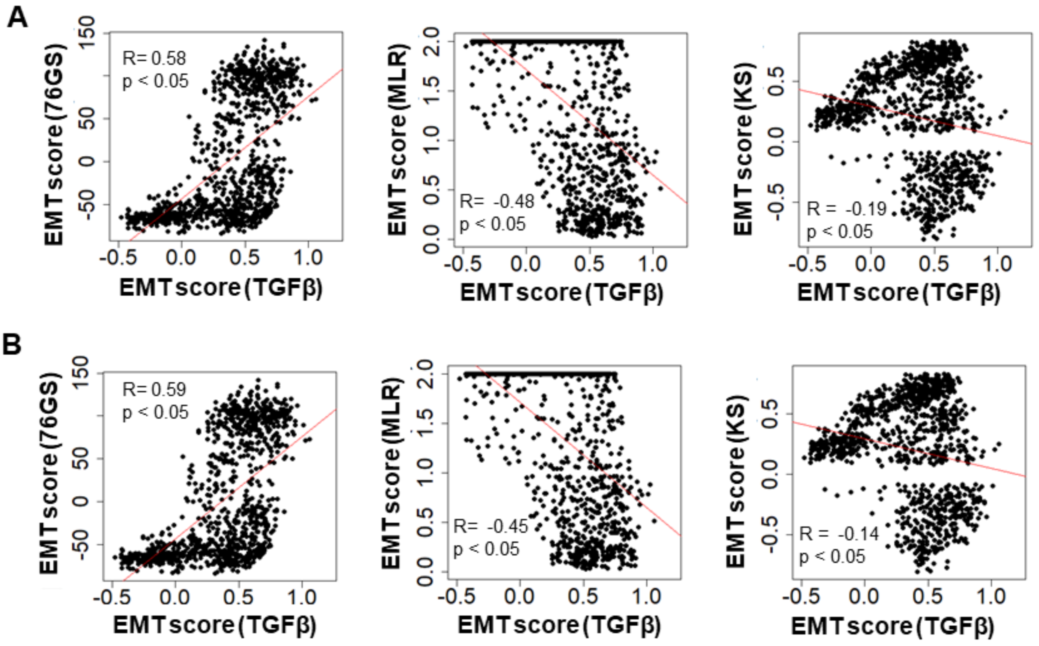


**Figure S3:** *EMT score correlation with TGFβ specific EMT scoring method in CCLE dataset(A) Pearson's correlation coefficient (B)Spearman's correlation. Correlation coefficient (R) and p-value (p) reported in each plot*

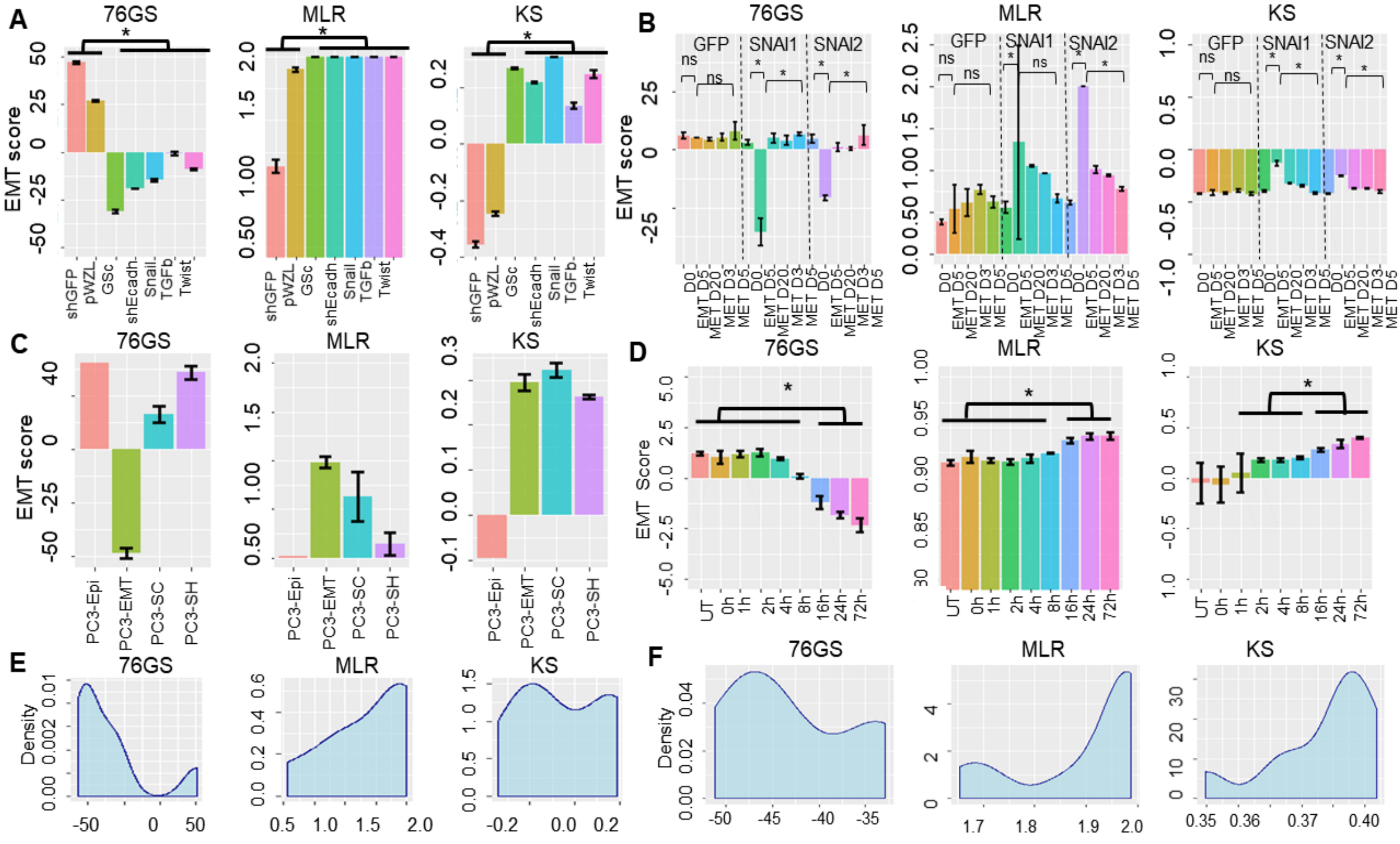


***Figure S4:*** *EMT scores of different EMT time series datasets (A) GSE24202- EMT induction by different EMT regulators (B) GSE84002- EMT and MET induction over time by GFP, SNAI1 and SNAI2. (C) GSE43489 -EMT/MET induction in PC3 cell line . (D) GSE17708 – EMT induction over time (E) GSE55470- CTCs from breast cancer patients (F) GSE50991- CTCs from ex vivo lung cancer model (* $p < 0.05$, n=3, two-tailed Student's t-test; error bars represent standard deviation for n =3). E and F represent kernel density plots.*

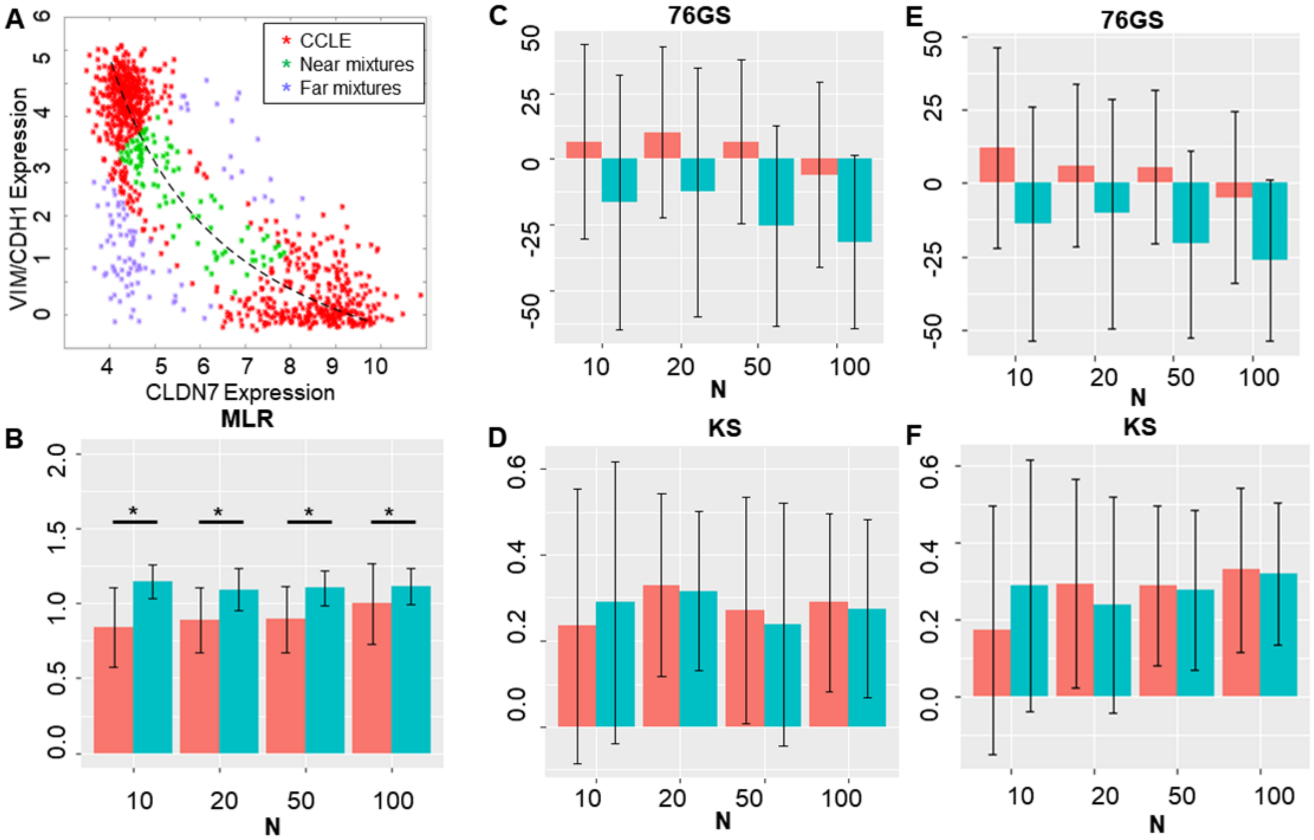


**Figure S5**: *(A) Scatter plot showing 100 farthest and closest samples based on the distance from mixture curve defined by median of 35 most pure E and pure M CCLE samples. (B) MLR EMT score for N(10,20,50,100) closest and farthest hybrid samples from median mixture curve. Bar plots showing EMT scores of N (10, 20, 50, 100) closest and farthest hybrid samples from mean mixture curve. (C) 76GS EMT score (D) KS EMT score. Bar plots showing EMT scores of N (10, 20, 50, 100) closest and farthest hybrid samples from median mixture curve. (E)76GS EMT score (F) KS EMT score. (* $p < 0.05$, N=10, 20, 50 & 100, two-tailed Student's t-test; error bars represent standard deviation for the given value of N)*
